# Birthweight Related Factors in Northwestern Iran: Using Quantile Regression Method

**DOI:** 10.5539/gjhs.v8n7p116

**Published:** 2015-11-18

**Authors:** Ramazan Fallah, Anoshirvan Kazemnejad, Farid Zayeri, Alireza Shoghli

**Affiliations:** 1 Department of Biostatistics, Faculty of Medical Sciences, Tarbiat Modares University, Tehran, Iran; 2 Department of Biostatistics, Faculty of Paramedical Sciences, Shahid Beheshti University of Medical Sciences, Tehran, Iran; 3 Department of Social Medicine, Zanjan Social Determinants of Health Research Center, Faculty of Medicine, Zanjan University of Medical Sciences, Zanjan, Iran

**Keywords:** birthweight, quantile regression, northwestern of Iran

## Abstract

**Introduction:**

Birthweight is one of the most important predicting indicators of the health status in adulthood. Having a balanced birthweight is one of the priorities of the health system in most of the industrial and developed countries. This indicator is used to assess the growth and health status of the infants. The aim of this study was to assess the birthweight of the neonates by using quantile regression in Zanjan province.

**Methods:**

This analytical descriptive study was carried out using pre-registered (March 2010 - March 2012) data of neonates in urban/rural health centers of Zanjan province using multiple-stage cluster sampling. Data were analyzed using multiple linear regressions andquantile regression method and SAS 9.2 statistical software.

**Results:**

From 8456 newborn baby, 4146 (49%) were female. The mean age of the mothers was 27.1±5.4 years. The mean birthweight of the neonates was 3104 ± 431 grams. Five hundred and seventy-three patients (6.8%) of the neonates were less than 2500 grams. In all quantiles, gestational age of neonates (p<0.05), weight and educational level of the mothers (p<0.05) showed a linear significant relationship with the i of the neonates. However, sex and birth rank of the neonates, mothers age, place of residence (urban/rural) and career were not significant in all quantiles (p>0.05).

**Conclusion:**

This study revealed the results of multiple linear regression and quantile regression were not identical. We strictly recommend the use of quantile regression when an asymmetric response variable or data with outliers is available.

## 1. Introduction

Birthweight is one of the main determinations for somatic and mental growth in infants and it is an important symptom of intrauterine growth (Alexander, Wingate, Mor, & Boulet, 2007). Birthweight is a multi-dimensional factor because numerous causes such as genetic, nutritional status of mothers, birth intervals and maternal care as well as social and economic environment are associated with it (Adam, Ismail, Nasr, Prins, & Smits, 2009). A weight over 2500 gram among at least 90% of neonates is one of the main aims of the WHO for developing countries. This aim is related to health and nutrition education during the pregnancy and also learning about prevention of repeated conception. An average estimate of the prevalence of low birthweight (a birthweight less than 2500 gram) in developed and developing countries are about 7% and 16%, respectively (Blanc & Wardlaw, 2005). In Iran, a prevalence rate of 7.2% was reported for low birthweight infants (Chaman et al., 2013).

Although the growth standards proposed by WHO can be regarded worldwide, the local conditions such as race, socio-economic status and other determinants were not considered to model the growth by this organization (Speiser et al., 2005). Therfore, the use of a country based growth evaluation tool in which the national standards of growth were applied can be more useful than the WHO approach (Abtahi, Doustmohammadian, & Abbdollahi, 2011). Several risk factors are associated with low birthweight. Although confirmed relationships were suggested for some predictors including maternal age and prematurity, the resultswere inconsistent for some othervariables, including maternal educational level (Chaman et al., 2013; Esimai & Ojofeitimi, 2014; Roudbari, Yaghmaei, & Soheili, 2007).

A literature review about the statistical methods for modeling the birthweight shows that the linear regression is the most common approach for determining the association between birthweight and related risk factors (Geraci, 2014; Quene & van den Bergh, 2004; Tian & Chen, 2006). However, this method is not the optimal approach due to some limitations such as requiring the normality assumption or homogeneity of variance of data and inability to manage properly the outlier values. In this context, the quantile regression, introduced by Knocker and Boast in 1978, is one of the methods that can overcome these limitations. This statistical modeling technique can be utilized to evaluate the association between the outcome and independent variables in each conditional quantile and hence is applicable for all data with a low, moderate and outlier values (Cade & Noon, 2003; Wei, Pere, Koenker, & He, 2006; Yu, Lu, & Stander, 2003).

There are a number of previously published manuscripts that used the quantile regression analysis to model the birthweight data worldwide; however, there are no publications in this context in Iran, Islamic Republic of Iran. Thus, we decided to conduct the present study in order to model the birthweight data on a large sample size (8456 newborns) using the quantile regression model as an appropriate statistical technique for analyzing this kind of data sets. The aim of this study was to determine the birthweight of the neonates and related factors in Zanjan province using the quantile regression method.

## 2. Materials and Methods

### 2.1 Study Population and Sampling

This cross sectional study was conducted in Zanjan province with a total population of 1020000 (49% of population were female). Zanjan province is located at the north west of Iran with 8 different districts with semi-mountainous climate. The target population was all the neonates in Zanjan province were born from March 2010 to March 2012. The study sample was selected by a multi-stage cluster sampling technique. In the first stage, four cities were randomly selected among eight cities of this province. In the second stage, thirty-two centers were randomly selected from eighty- two governmental urban and rural health centers. The rural health centers typically cover more than 98% of pregnant women while urban health centers coverage is about 75%. At the next step, all the health files of neonates under two years in these thirty-two centers were evaluated. Consequently, a total of 8,456 records were included in the study. In the present study, since the researchers had a variety of descriptive and analytical purposes (including estimating the mean birthweight, determining indicators of birthweight and etc.), a large sample size theory was used to ignore the sample size formula. To do this, we selected 25% of the existing files in these centers (8456 from about 34000 files) over two years period.

### 2.2 Data Collection and Variables Under Study

In this research, neonate’s birthweight was considered as the main outcome (dependent variable) in the study. For each infant, the birthweight was extracted based on existing data in the health files. Their health files information was collected by experienced health workers. For instance, the infant’s weight was measured using regular calibrated and standardized baby scales (Seca, Germany) with an accuracy of 10 grams in these centers. In addition, the place of residence (urban or rural), neonate’s sex(male or female), mothers educational level (illiterate, primary, secondary school, diploma, academy) maternal weight (kg), height (cm), job status (Homemaker, employee), maternal age (year), parity (No of 24 weeks or more born babies alive or still birth), and gestational age (week) were considered as the potential indicators (independent variables) for the birthweight in modeling process. Data were gathered by a predesigned checklist which was filled out based on registered data in health records of neonates.

### 2.3 Inclusion and Exclusion Criteria

All under 24 months babies at the time of the data collection were eligible to enter to the study. The babies with the history of any known genetic or congenital disorders, any mental or physical disability and incomplete record due to interruption in the continuum of the care were discarded from the study. Therefore, 694 files were discarded from 9150 files and 8456 were included in the study.

### 2.4 Statistical Analysis

In this study, the quantile regression method was used for assessing the effect of different indicators on birthweight. In quantile regression, the weighted sum of the absolute deviations of the error term was minimized to estimate the related parameters instead of minimizing the sum of the squared residual in ordinary regression model. When we have a response variable with highly skewed distribution, quantile regression can be properly used to model the tails of it. For a random sample {*y*_1_, *y*_2_,…, *y_n_*} of *Y*, it is well known generally the sample quantile, Ψ(τ) which is considered an analogue to *Q*(τ), can be formulated as the solution of the optimization problem, i.e.


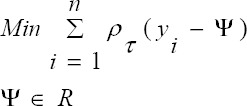


The linear conditional quantile function *Q(τ)│X = x) = x′β(τ)*, can be estimated by solving


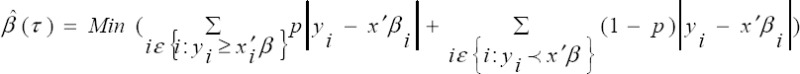


For any quantile τ ∈ (0,1) The quantity β^(τ) is called the τth regression quantile (Koenker & Hallock, 2001; Yu et al., 2003). To fit this model; the SAS software version 9.2 was utilized. A p-value less than 0.05 were considered statistically significant.

#### 2.5 Ethical Considerations

All aspects of the study protocol were authorized by the Research Ethics Committee of Tarbiat Modares University in Tehran, prior to the initiation of this study. This study was conducted based on registered data in the health centers and all related information of the participants will be kept confidential.

### 3. Results

A total of 8456 neonates, 4146 were female (49.0%). From maternal education aspect, 449 (5.3%) mothers were illiterate, 2349 (27.8%) had the primary school degree, 1909 (22.6%) secondary school, 1941(22.9%) diploma and 1688 (20.0%) academic degree. A number of 1167 (13.8%) mothers were employed and 7289 (86.2%) were homemaker. Of 8456 mothers, 4245 (50.2%) lived in rural areas (Table 1). The mean ± SD of mother’s weight, height, age and infant’s gestational age was respectively, 27.1±5.4, 62.2±9.4, 158.5±5.6, and 38.6±1.3.

**Table 1:** Univariate results for assessing the effect of different factors on birthweight

Characteristics	Category	Mean(gram)	SD*
Mother’s education	Illiterate	3086.46	491.6
	Primary school	3091.19	431.8
	Secondary school	3109.28	427.3
	Diploma	3114.09	428.2
	Higher education	3107.55	426.9
Place of residence	Urban	3091.43	438.0
	Rural	3116.68	424.8
Mother’s job	Homemaker	3107.57	432.9
	Employee	3082.83	423.9
Neonate’s gender	Boy	3123.81	443.8
	Girl	3083.63	417.9

* Standard Deviation.

Among the neonates under study, 573 (6.8%) had a weight less than 2500 grams. The results of multiple linear regression analysis for assessing the relationship between birthweight and different indicators including place of residency, neonatal sex, maternal educational level, maternal job status, maternal weight, maternal height, parity and gestational age were displayed in Table 2. These results shows that, controlling for other explanatory variables, the rural neonates had significantly higher weight (36.6 gram) than the urban neonates (p<0.001). These results shows that, after controlling for the effects of the explanatory variables, the male neonates were significantly heavier (41.4 gram) than the female neonates (p=0.001). In multiple linear regressions the birthweight of the neonates whose mothers had academic educational was significantly different other (p<0.05).

**Table 2:** Multiple linearregression results for assessing the relationship between different indicators and birthweight

Characteristics	Category	B*	SE**	95%CI***		p-value

				LL	UL
Place of residence	Rural	36.56	10.54	15.89	57.23	0.001
	Urban		Reference			
Neonate’s gender	Girl	-41.38	9.18	-59.38	-23.39	0.001
	Boy		Reference			
Mother’s Education	Illiterate	-89.54	26.59	-141.67	-37.42	0.001
	Primary school	-92.39	19.17	-129.99	-54.80	0.001
	Secondary school	-50.72	19.23	-88.31	-13.14	0.008
	Diploma	-36.34	18.17	-71.86	-0.82	0.045
	Higher education		Reference			
Mother’s job	Homemaker	-69.41	19.13	-106.92	-31.91	0.001
	Employee		Reference			
Mother’s age		2.44	1.17	0.15	4.73	0.037
Parity		-12.67	6.85	-26.09	0.75	0.064
Mother’s weight		5.70	0.55	4.63	6.77	0.001
Mother’s height		3.47	0.88	1.75	5.20	0.001
Gestational age		76.82	3.65	69.67	83.98	0.001

* Estimate of the model parameter;

** Standard error of the estimate;

*** CI=Confidence Interval; LL=lower limit, UL=upper limit.

Moreover, the neonates of employed mothers had significantly higher weight (69.41gram) than the neonate with unemployed r mothers (p=0.001). A significant association was also obtained between the gestational age and birthweight, so that the birthweight was increased 76.8 gram with one week increasing in gestational age. In addition, a significant relationship between the maternal age and birthweight was found, so the neonatal weight increased 2.4 gram with one year increasing in maternal age (p=0.037).

**Figure 1:**
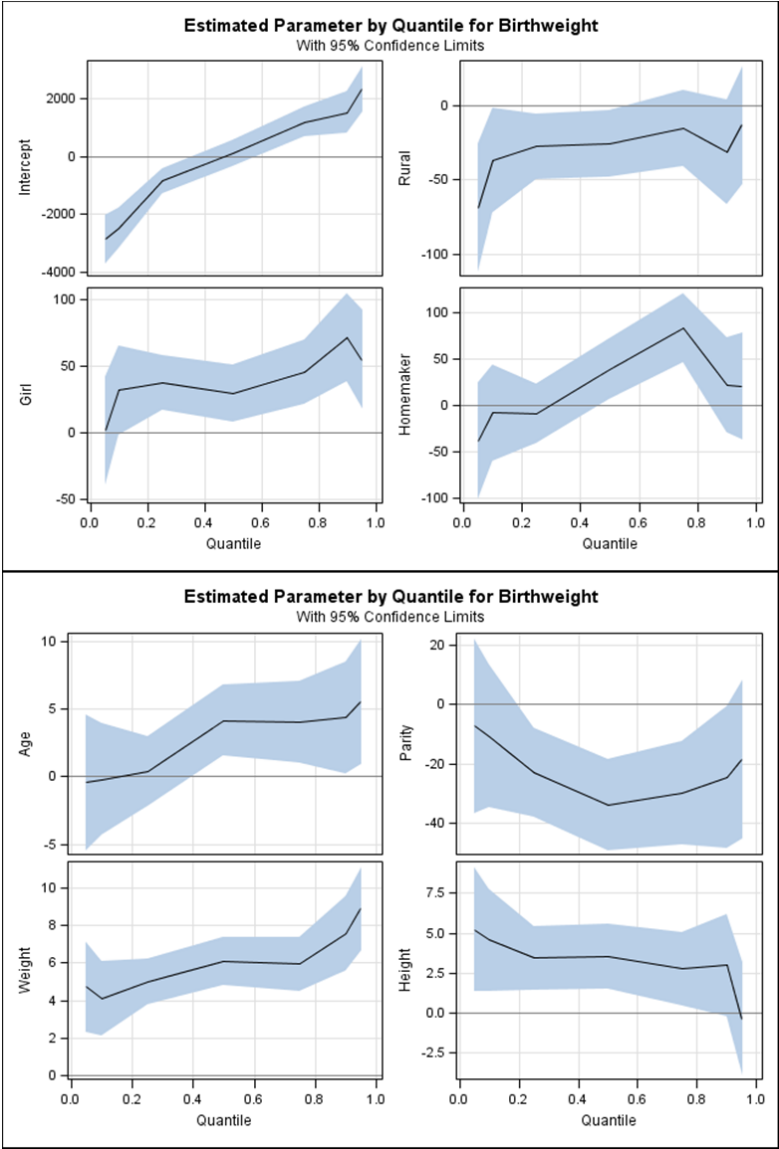
Association between birthweight and residence place (rural relative to urban), neonatal gender (girls relative to boys), mother’s job (homemaker relative to employee), parity, mother’s weight and mother’s height in the different quantiles

As mentioned before, we also fitted a quantile regression model to the birthweight data. The obtained results were shown in Table 3. The quantile regression coefficients for place residece, controlling for other independent variables, had different behavioral in different quantiles. In quantile 5 for instance, the weight of rural neonates was higher than the urban neonate 96.5 gram (p<0.001). However, in upper quantiles, a less difference was found, so that the difference was not significant in quantile 95. For gestational age, the results showed that one week increasing in gestational age increased the birthweight about 112.6 gram in quantile 5 (p<0.001), while in upper quantiles the association was different, so that in quantile 95 one week increasing in gestational age increased the birthweight only 23.1 gram. We found no significant association between the maternal age and birthweight in lower quantiles; however the association was significant in upper quantiles. For example, in quantile 5, one year increasing in maternal age led to a non-significant increase in birthweight (only 0.9 gram), while in quantile 95 increasing in maternal age led to a significant increase in birthweight (5.3 gram), (p=0.013). Similar results were obtained for other indicators. In quantile regression a significant difference in neonatal birthweight ofinfants who had illiterate mothers was shown with those in the reference category (newborns whosemothers had academic level of education) in quantiles 5 (p=0.001), 10 (p=0.002) and 25 (p=0.001), while for neonates whose mothers had primary school education the difference was significant in all quantiles (p<0.05) except for quantile 50 (p=0.057). For infants whose mothers had secondary school education, a significant difference was found only inquantile 5 (p=0.040). However differences were not significant in any quantiles for mothers with diploma. More details are reported in Table 3 and Figure 1.

**Table 3:** Quantile regression results for assessing the relationship between different indicators and birthweight

Quantiles		5	10	25	50	75	90	95
		B*	B	B	B	B	B	B
Characteristics	Category	SE**	SE	SE	SE	SE	SE	SE
		p-value	p-value	p-value	p-value	p-value	p-value	p-value

		96.5	79.3	15.5	38.4	27.9	39.8	26.9
Place of residence	Rural	22.9	20.2	12.9	12.7	13.9	20.1	18.9
		0.001	0.001	0.001	0.003	0.442	0.482	0.156
	Urban			Reference				
		-3.3	-24.7	-41.9	-35.6	-41.9	-62.6 17.6	-61.8 16.5
Neonates gender	Girl	19.9	17.6	11.2	1.1	12.1	0.001	0.002
		0.87	0.16	0.001	0.001	0.001		
	Boy			Reference				
		-189.0 57.6	-154.4	-140.6 32.3	-21.9	-59.3	-73.4 50.6	21.5
Mother’s Education	Illiterate	0.001	50.7	0.001	32	34.9	0.147	47.5
			0.002		0.493	0.809		0.652
	Primary school	-160.6 41.7	-51.7	-93.8	-44.2	-60.5	-87.2	-93.6
		0.001	36.7	23.4	23.9	25.9	36.9	34.5
			0.001	0.001	0.057	0.017	0.018	0.007
	Secondary	-81.9	-69.5	-30.3	-25.3	-32.6	-57.8	-42.7
	school	41.7	36.7	23.4	23.2	25.2	36.7	35.5
		0.04	0.059	0.196	0.276	0.196	0.116	0.216
	Diploma	-75	-53.9	-29.4	2.1	-16.1	-42.5	-61.4
		39.7	34.7	22.1	21.9	23.8	34.7	32.6
		0.057	0.12	0.183	0.924	0.498	0.22	0.059
	Higher education			Reference				
		-80.9	-47.7	-27.1	-47.7	-109.3	-73.7	-64
Mother’s job	Homemaker	41.6	36.6	23.3	23.1	25.2	6.6	34.4
		0.517	0.193	0.245	0.039	0.001	0.044	0.063
	Employee			Reference				
		-0.9	0.2	0.9	3.9	4.3	4.1	5.3
Mother’s age		0.6	2.3	1.4	1.4	1.5	2.2	2.1
		0.735	0.927	0.519	0.006	0.005	0.065	0.013
Parity		10	5.8	-5.6	-27.8	-24.8	-28.4	-23.3
		5.9	14	8.9	8.8	9.6	14	13.2
		0.531	0.677	0.533	0.002	0.01	0.042	0.068
		5.0	3.5	4.9	6.1	6	7.9	8.7
Mother’s weight		1.9	1.1	0.7	0.7	0.7	1	13.2
		0.001	0.001	0.001	0.001	0.001	0.001	0.001
		4.2	4.5	3.1	3.5	3	2.1	-0.13
Mother’s height		1.9	1.7	1.1	1.1	1.2	1.7	0.6
		0.029	0.001	0.003	0.001	0.01	0.205	0.936
		112.6	107.3	76.9	55.1	38.4	28.3	23.2
Gestational age		7.9	7	4.4	4.4	4.8	7	6.6
		0.001	0.001	0.001	0.001	0.001	0.001	0.004

* Estimate of the model parameter.

** Standard error of the estimate.

## 4. Discussion

Growth is considered as one of the fundamental health problems closely related to environmental, economical, genetic and individual lifestyle factors. As mentioned in the results section, the mean birthweight was 3104 ±431 in our sample, while in other studies in Iran it has been reported 3248±453 in Qazvin 3166±435 in Tehran and 3272±389 in Gilan (Alizadeh & Delbari, 2012; Chaman et al., 2013; Mahmoodi et al., 2013). In addition a study in Denmark showed the mean ± SD birthweight of 3413±545 in female newborns and 3533±579 in males (McGrath et al., 2007). The birthweight in two other studies in north Ethiopi and Palestine were 3282±591, 3188±461respectively (Halileh, Abu-Rmeileh, Watt, Spencer, & Gordon, 2008; Teklehaimanot, Hailu, & Assefa, 2014). In all above studies the mean birthweight of infants was more than our study. However a lower mean birthweight was reported by Zeleke et al. (2972±5 476) (Zeleke, Zelalem, & Mohammed, 2012). Differences in birthweight estimates between different studies can be mainly attributed to differences in genetic, socioeconomic and lifestyle factors, also the type of sampling and statistical analysis applied.

The prevalence of low birthweight infants (birthweight ≤ 2500 grams) was 6.8% in our study. While this proportion was 5.7% among Spanish and 3.2% among Sweden neonates (Moser, Li, & Power, 2003). The prevalence of low birthweight infants in several studies were higher than our study i.e.7.6% among American (Moser et al., 2003) 8.5% in Japan (Morisaki, Esplin, Varner, Henry, & Oken, 2013) and 17.1% in Ethiopia (Zeleke et al., 2012). Low birthweight babies are indicative of the quantity and quality of maternal health services in the country and the mothers` nutritional status. So as expected this indicator show better figures in developed countries.

In this study, we also found higher mean birthweight in rural infants than urban infants. Similar to our study, Zeleke et al obtained a significant association between birthweight and residency (Zeleke et al., 2012), however Halileh et al did not find such a relationship (Halileh et al., 2008). This slightly but significant difference can be attributed to more rigorous maternal care and health education activities in rural area as a result of a better coverage of primary health care in these areas compared to urban areas.

In line with other studies, we also obtained a significant association between increasing maternal age and increasing birthweight of the infants (Dollar, 2005; Khadem et al., 2012; Mahmoodi et al., 2013; Muula, Siziya, & Rudatsikira, 2011; Sutan, Mohtar, Mahat, & Tamil, 2014). However, this relationship was not found in Halileh et al study (Halileh et al., 2008).

We obtained a significant association between maternal education level and birthweight, so that a higher birthweight obtained among the neonates whose mothers had higher level of education. Other studies showe dinconsistent findings i.e. some studies revealed positive association (Brown, Yelland, Sutherland, Baghurst, & Robinson, 2011; Hamta, Khalilian, Farhadi, & Ranjbaran, 2013; Hoffman et al., 2007; Khadem et al., 2012; Li, Graubard, & Korn, 2010), while this relation was not found in some other studies (Halileh et al., 2008; Mahmoodi et al., 2013; Muula et al., 2011). It might be expected that educated mothers have a better knowledge of standard motherhood and may be have a better economic status compared to those uneducated.

In line with a lot of similar studies, our study showed a significant relationship between the gestational age (Chhabra, Sharma, Grover, & Aggarwal, 2004; Morisaki et al., 2013; Teklehaimanot et al., 2014; Zeleke et al., 2012), and mothers height and weight with birthweight (Khadem et al., 2012; Mahmoodi et al., 2013; Sutan et al., 2014).

We used quintile regression to analyze the birthweight data. Due to an unavoidable outlier data structure in birthweight, we cannot properly determine the associations in all parts of outcome distribution using the classic linear regression model; in contrast the quantile regression enables us to provide such an opportunity. For instance, a significant association between residency and birthweight in all parts of outcome distribution was observed in linear regression, while in quantile regression the association was significant only among (first 50% of neonate weight rank) the low birthweight neonates.

The other benefit of the quantile regression is its ability to analyze the relationship between to variables more accurately. Our findings based on linear regression showed no significant relationship between neonatal birthweight and parity, whereas the quantile regression resulted in different finding. Using this model, we found that this relationship is significant in quantile 50 and over.

As discussed above, in this study the quantile regression resulted in the following findings: indirect association between the maternal education level and birthweight in lower quantile, but there was no such association in higher quantiles. Our study showed a significant relationship between the gestational age and birthweight, particularly in lower quantile. Based on our results, the mean weight of neonates with mothers who had secondary school educational level was only 81.9 grams (but significant) less than neonates with mothers who had academic level in the lowest quantile. The ordinary linear regression shows the association between a response variable and different indicators in an average form, while the quantil regression shows more detailed result for the association between variables or differences between categories. In other words, the quantil regression can be thought as using ordinary linear regression in different quantiles of the response variable.

Therefore, for an independent variable the quantile regression results may be significant in some quantiles and non-significant in other quantiles. Although a significant association was obtained between maternal occupation status and birthweight in the linear regression, the association was significant only in upper quantiles.

Our study had some limitations; we conducted a retrospective study in which only the existing data of the infants in primary health care centers were used, however we are almost assured all infants in a rural area have reliable data at least for the first 24 month of life.

## 5. Conclusion

Our findings confirm the current knowledge about relationship between birthweight and some known factors discussed above. However external factors like mothers education, place of residency still remain a mystery. As different studies show different result, we suggest additional studies to more investigate the relationship between birthweight and factors like mothers educational level, place of residence, socioeconomic status etc.
